# Interfacial‐Polarization Engineering in BNT‐Based Bulk Ceramics for Ultrahigh Energy‐Storage Density

**DOI:** 10.1002/advs.202409113

**Published:** 2024-11-08

**Authors:** Wenjun Cao, Li Li, Kun Chen, Xuecen Huang, Feng Li, Chunchang Wang, Jun Zheng, Xu Hou, Zhenxiang Cheng

**Affiliations:** ^1^ Laboratory of Dielectric Functional Materials School of Materials Science & Engineering Anhui University Hefei 230601 China; ^2^ School of Material and Chemical Engineering Chuzhou University Chuzhou 239000 China; ^3^ Institute of Physical Science and Information Technology Anhui University Hefei 230601 China; ^4^ Department of Industrial and Systems Engineering Research Institute for Advanced Manufacturing Hong Kong Polytechnic University Hung Hom Kowloon, Hong Kong 999077 China; ^5^ Hong Kong Polytechnic University Shenzhen Research Institute Shenzhen 518060 China; ^6^ Institute for Superconducting and Electronic Materials Faculty of Engineering and Information Sciences University of Wollongong North Wollongong NSW 2500 Australia

**Keywords:** breakdown strength, energy storage, interfacial‐polarization engineering, lead‐free ceramics, relaxor ferroelectrics

## Abstract

Ceramic capacitors, known for their exceptional energy‐storage performance (ESP), are crucial components in high‐pulsed power systems. However, their ESP is significantly constrained by breakdown strength (*E*_b_), which is influenced by interfacial polarization. This study delves into the physics, characterization, and application of interfacial polarization. The findings indicate that key factors affecting ESP, such as grain size, relaxor factor, and bandgap, are intrinsically linked to interfacial polarization, establishing it as the most critical determinant of ESP. To demonstrate the practical applications of interfacial polarization engineering, lead‐free ceramics of (1‐x)(0.94Bi_0.5_Na_0.5_TiO_3_‐0.06BaTiO_3_)‐*x*Ca_0.7_Bi_0.2_(Sn_0.5_Ti_0.5_)O_3_ (abbreviated as (BNT‐BT)‐*x*CBST is designed, where *x* = 0, 0.1, 0.15, 0.2, and 0.25). The (BNT‐BT)‐0.25CBST sample, with a thickness of 120 µm, achieved an ultrahigh recoverable energy‐storage density (*W*_rec_) of 12.2 J cm^−3^ and a high efficient (η) of 88.8%, along with excellent temperature/frequency stability and outstanding charge/discharge performance. The remarkable ESP is attributed to the suppression of interfacial polarization, which significantly enhances *E*_b_. This work highlights the pivotal role of interfacial polarization engineering in the development of energy‐storage ceramics with superior comprehensive performance.

## Graphical abstract

In this work, we addressed the key issuses of physics, characterization, and application related to interfacial polarization, establishing which as the most critical determinant of dielectric ceramic energy‐storage performance (ESP). Superior ESP of an ultrahigh recoverable energy‐storage density of 12.2 J/cm^3^ and a high efficient of 88.8% were achieved in Bi_0.5_Na_0.5_TiO_3_ system by interfacial polarization engineering.

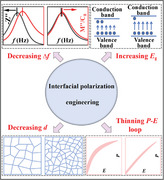

## 1 Introduction

Ceramic capacitors play a crucial role as energy storage components in integrated electronic systems due to their ultra‐high power density, ultrafast discharge rate, and excellent stability.^[^ ^1^, ^2^ ^]^ Among various dielectric materials, inorganic ceramics stand out due to their good thermal and chemical stability, long service life, low cost, and high mechanical strength.^[^ ^3^ ^]^ Consequently, extensive research has been conducted on ceramics in the forms of thin films (<1 µm),^[^ ^4^, ^5^ ^]^ thick films (1–100 µm),^[^ ^6^, ^7^ ^]^ multilayer films,^[^ ^8^, ^9^ ^]^ and bulks (>100 µm).^[^ ^10^, ^11^ ^]^ Unfortunately, the recoverable energy‐storage density (*W*_rec_), of ceramic materials is relatively low (<5 J cm^−3^). Although optimization methods such as chemical doping,^[^ ^12^, ^13^ ^]^ secondary structure modification,^[^ ^14^, ^15^ ^]^ and advanced material fabrication technologies^[^ ^16^, ^17^ ^]^ can significantly increase the *W*_rec_ value to 6–10 J cm^−3^, achieving ultrahigh *W*_rec_ exceeding 10 J cm^−3^ remains rare. Table S1 (Supporting Information) lists recent reports of *W*_rec_ >10 J cm^−3^, showing that the majority of samples with ultrahigh energy storage density have a thickness below 100 µm, classifying them as thick films. Reducing the sample thickness can increase the *W*_rec_ value but does not enhance the total energy storage amount. Therefore, achieving an ultrahigh *W*_rec_ value and efficiency (*η* > 80%) in bulk lead‐free ceramic capacitors is a significant challenge.

Theoretically, the *W*_rec_ value is determined by two factors: the polarization strength (Δ*P* = *P*_m_ − *P*_r_, with *P*_m_ and *P*_r_ being the maximum and remnant polarizations, respectively) and the breakdown strength (*E*_b_, the maximum electric field that the material can withstand).^[^ ^18^ ^]^ Unfortunately, there is a trade‐off between these two factors‐it is challenging to achieve large values for both simultaneously.^[^ ^19^ ^]^ Since the *W*_rec_ value, in the case of linear dielectrics, is quadratically related to *E*_b_, it is particularly important to boost the *W*_rec_ value by improving the breakdown strength. So far, all the ultrahigh *W*_rec_ values reported in the literature have been obtained under high fields (>30 kV mm^−1^, see Table S1, Supporting Information). Apart from the intrinsic factor of the bandgap, the breakdown strength is greatly influenced by many extrinsic factors, including various defects (second phase, porosity, space charge, etc.) and geometric parameters (grain size, sample thickness, and electrode size).^[^ ^1^ ^]^ Many methods, as mentioned above, have been proposed to modulate these extrinsic factors to improve *E*_b_. Very recently, we have identified the interfacial polarization strategy‐performed by reducing space charge concentration and modifying field redistribution‐ as a powerful strategy to boost *E*_b_. As a result, an ultrahigh *W*_rec_ value of 15.1 J cm^−3^ and a high *η* of 82.4% was achieved in bulk 0.62(0.94Bi_0.5_Na_0.5_TiO_3_‐0.06BaTiO_3_)‐0.38Ca_0.7_La_0.2_TiO_3_ ceramic with a thickness of 120 µm.^[^ ^20^ ^]^ In addition to the Bi_0.5_Na_0.5_TiO_3_ system, the interfacial polarization strategy has been documented to be applicable in NaNbO_3_,^[^ ^21^ ^]^ BaTiO_3_,^[^ ^22^ ^]^ and even linear dielectric systems,^[^ ^23^ ^]^ indicating that the strategy has very good universality. Although the interfacial polarization strategy successfully addresses the challenge of achieving *W*_rec_ > 10 J cm^−3^ in bulk ceramic systems, several important questions remain unanswered: 1) How can we physically understand that inhibiting interfacial polarization leads to the enhancement of *E*_b_? 2) How can we accurately characterize interfacial polarization? 3) How do we choose the appropriate modifiers to regulate interfacial polarization?

**Figure 1:**
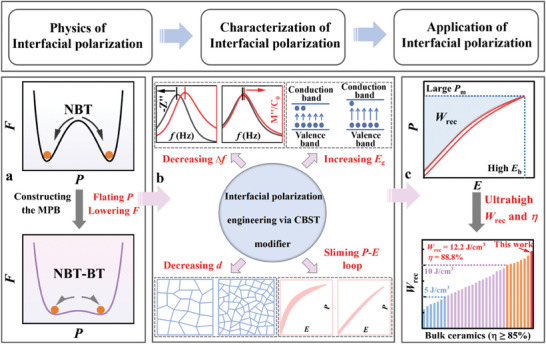
Schematic diagram of the interfacial polarization engineering. a) constructing MPB for easy polarization flipping. b) suppressing interfacial polarization for increased *E*_b_. c) improved ESP.

In this work, we present a theoretical investigation of the physics and characterization of interfacial polarization. Guided by the theoretical results, the application of interfacial polarization was systematically studied in Bi_0.5_Na_0.5_TiO_3_ (BNT) system. BNT was used as a model system because 1) it is a canonical system for energy‐storage applications^[^ ^24^ ^]^ and 2) BNT‐based ceramics have rarely been reported to achieve both ultrahigh *W*_rec_ (>10 J cm^−3^) (Table S1, Supporting Information). To achieve ultrahigh *W*_rec_ in this system, BaTiO_3_ (BT) was judiciously introduced to form a morphotropic phase boundary (MPB) in BNT‐BT solid solution with the purpose of reducing polarization flipping energy for a smaller *P*_r_ value (**Figure** **1**a). Linear‐like dielectric Ca_0.7_Bi_0.2_(Sn_0.5_Ti_0.5_)O_3_ (CBST) was used as a modifier aiming at modulating interfacial polarization, phase structure, and domain structure. The modifier contains large *E*_g_ oxides of CaTiO_3_ (3.4 eV) and CaSnO_3_ (4.2 eV).^[^ ^25^, ^26^ ^]^ The compositing of these highly insulating oxides can not only increase the forbidden bandwidth of the solid solution but also enhance grain resistance, which, in turn, reduces the resistance mismatch between grain and grain boundary. So it is reasonable to suppress interfacial polarization. Meanwhile, linear dielectrics can dilute ferroelectric long programs and hinder grain growth, thus reducing grain size, and regulating phase and domain structures (Figure 1b).^[^ ^27^ ^]^ Hence, (1‐*x*)(0.94Bi_0.5_Na_0.5_TiO_3_‐0.06BaTiO_3_)‐*x*Ca_0.7_Bi_0.2_(Sn_0.5_Ti_0.5_)O_3_ with *x* = 0, 0.1, 0.15, 0.2, and 0.25, abbreviated as (BNT‐BT)‐*x*CBST, were designed. Their energy storage properties were systematically investigated. Encouragingly, the (BNT‐BT)‐0.25CBST ceramic with the weakest interfacial polarization shows an ultrahigh *W*_rec_ of 12.2 J cm^−3^ and a high η of 88.8% (Figure 1c). This work establishes interfacial polarization engineering as a powerful approach for designing dielectric ceramics for outstanding ESP.

## 2 Results and Discussion

### 2.1 The Physics of Interfacial Polarization

**Figure 2:**
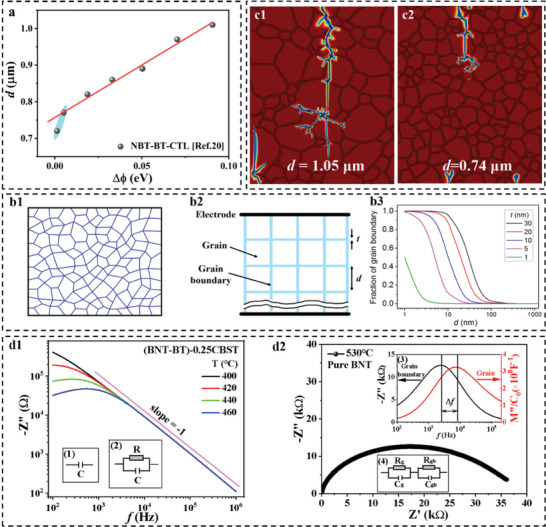
The physics of interfacial polarization. a) The relationship between the mean grain size *d* and the strength of the interfacial polarization (Δϕ) found in BNT system. Here, CLT = Ca_0.7_La_0.2_TiO_3_, b1) and b2) Sketches of actual and idealized structures of a ceramic sample, respectively, and b3 the deduced grain‐boundary fraction as a function of *d*. c1) and c2) COMSOL simulations of the breakdown path of the samples with *d* = 1.05 and 0.74 µm, respectively. d1) The impedance spectroscopic plots of the (BNT‐BT)‐0.25CBST sample recorded at different temperatures. Insets show the equivalent circuits of an ideal (1) and leaky (2) capacitors. d2) Complex impedance plot of the pure BNT sample measured at 530 °C. Inset (3) is the corresponding impedance and modulus spectroscopic plots. Inset (4) shows the equivalent circuit for both grain and grain boundary.

Many studies have shown that reducing the average grain size enhances *E*_b_,^[^ ^20^ ^]^ similar to the effect of interfacial polarization. Therefore, it implies there might be a common physics underlying both factors. To corroborate this inference, **Figure** **2**a shows the average grain size *d* as a function of Δφ, the activation energy difference between grain and grain boundary resistances introduced to characterize the strength of interfacial polarization. A perfect linear relationship between *d* and Δφ is observed, confirming that the underlying physics of refining grain size is actually suppressing interfacial polarization. This can be understood by the fact that the grain growth of ceramic materials during high‐temperature sintering mainly depends on two factors: thermally assisted ion migration and built‐in field‐assisted ion migration. The built‐in field originates from the space charge accumulated at the grain‐grain boundary interfaces. Without the space charge, i.e., when interfacial polarization disappears, grain growth loses an important driving force, thereby strongly limiting the grain growth. It is worth emphasizing that, as highlighted by the yellow oval, the *d* values suddenly plummet as Δφ trends to zero. Correspondingly, a step‐like increase in *E*_b_ occurs, as confirmed in Ref.[20] This significantly boosted *E*_b_ is not solely the consequence of the sharp decrease in grain size. To determine the real reason for the step‐like increase in *E*_b_, the brick‐and‐mortar structure for ceramics (Figure 2b1) is simplified to a manageable model as sketched in Figure 2b2, where the grains are cubes of uniform side *d* surrounded by the grain boundaries of uniform thickness *t*.^[^ ^28^ ^]^ The volume fraction of grain boundaries (*f*_gb_) is then

(1) $$f_{\mathrm{gb}}=1-\frac{1}{{\left(1+t/d\right)}^{3}}$$

As shown in Figure 2b3, fixed *t*, *f*_gb_ significantly increases when *d* decreases to a magnitude comparable with *t*. This behavior resembles percolation. When the volume fraction approaches the percolation threshold, the dielectric strength increases exponentially and undergoes a sudden change at the percolation threshold.^[^ ^29^ ^]^ Although this needs to be explicitly studied by percolation theory, the *E*_b_ enhancement can be theoretically addressed by COMSOL simulation. Figure 2c1,2 shows the COMSOL simulation results of the electric tree for the samples with *d* = 1.05 and 0.74 µm, respectively. Notably, the electric tree for the sample with *d* = 1.05 µm shows a clear main trunk crossing the grains, resulting in the formation of a conductive filament, i.e., a breakdown path. In contrast, for the sample with *d* = 0.74 µm, the electric tree is impeded by the grain boundaries, leading to many shunted branches without a main trunk. Therefore, the breakdown path is difficult to form in this sample.

### 2.2 The Characterization of Interfacial Polarization

We now turn our attention to the second question. As we all know, interfacial polarization is active in the low‐frequency range. The interfacial polarization relaxation peak gradually decreases as the measurement temperature increases. In our previous paper, we deduced a general relationship between the peak intensity (Δε″) and the measurement temperature (*T*) as follows:^[^ ^30^ ^]^

(2) $$\Delta \varepsilon^{\prime \prime }=\frac{A}{B+C\exp(\Delta \varphi /k_{B}T)}$$

where *A*, *B*, and *C* are constants and Δφ is the difference in activation energy between grain and grain boundary resistances. This relationship indicates that Δφ could be the best parameter for characterizing interfacial polarization. However, extracting the Δφ parameter requires data fitting on the complex impedance spectrum (CIS) based on equivalent circuits. Different researchers use different equivalent circuits, which can lead to significant errors. Therefore, an accurate criterion to characterize interfacial polarization is imperative. Energy‐storage dielectric materials are typical insulating materials. In the case of negligible leakage current (e.g., insufficient low‐temperature range), the ceramic capacitor can be modeled by the equivalent circuit of an ideal capacitor (inset (1) in Figure 2d1). The impedance is given by:

(3) $$Z*=Z^{\prime }-jZ^{\prime \prime }=\frac{1}{j\omega C}$$

where *j* ^2^ = −1, ω is the angular frequency and *C* is the capacitance. The imaginary part of the impedance is:

(4) $$Z^{\prime \prime }=-\frac{1}{\omega C}$$

Therefore, a perfectly straight line with a slope of ‐1 could be obtained in a bilogarithmic plot as confirmed in Figure 2d1. The impedance spectroscopic plots of the (BNT‐BT)‐0.25CBST sample were measured at different temperatures. The curve recorded at 400 °C exhibits the linear feature except for the deviation in the low‐frequency range. As the measurement temperature increases, the deviation becomes notable and the curve shows a peak, indicating that the leakage current cannot be neglected, and the capacitor behaves as a leaky capacitor, which can be modeled by an *R*‐*C* circuit (inset (2) in Figure 2d1). The resistor (*R*) is introduced to describe the leakage current. In this case, the imaginary parts of impedance and electric modulus (M* = *j*ω*C*_0_ Z*, where *C*_0_ is the vacuum capacitance of the measuring cell) are given by:^[^ ^31^ ^]^

(5) $$Z^{\prime \prime }=R\left[\frac{\omega \tau }{1+{\left(\omega \tau \right)}^{2}}\right]$$

(6) $$M^{\prime \prime }=\frac{C_{0}}{C}\left[\frac{\omega \tau }{1+{\left(\omega \tau \right)}^{2}}\right]$$

where τ = *RC* is the relaxation time. Based on these equations, the spectroscopic plots of $Z^{\prime \prime }$ and $M^{\prime \prime }$ should register peaks at the same frequency where ωτ = 1. Meanwhile, a semicircle can be found in the CIS. However, the CIS usually exhibits a depressed semicircle, as shown in Figure 2d2, where the CIS of the pure BNT sample is recorded at 530 °C. The spectroscopic plots of $Z^{\prime \prime }$ and $M^{\prime \prime }$ reveal that the $Z^{\prime \prime }$ and $M^{\prime \prime }$ peaks are distinctly separated (inset (3) in Figure 2d2). This finding implies that the CIS is contributed by two *R*‐*C* circuits connected in series (inset (4) in Figure 2d2), with one representing the contribution from grains and the other from the grain boundaries. Equations (5) and (6) show that the $Z^{\prime \prime }$ peak emphasizes the contribution of large resistance, while the $M^{\prime \prime }$ peak highlights the contribution of small capacitance. Since the grain boundary is considered as a defective phase with a smaller thickness than that of the grain, the resistance, and capacitance of the grain boundary are much larger than those of the grain.^[^ ^32^ ^]^ Therefore, the $Z^{\prime \prime }$ peak represents the grain boundary information, while the $M^{\prime \prime }$ peak reflects the grain information. By combining the impedance and modulus spectra, the contribution of grains and grain boundaries can be effectively separated without any equivalent circuits. When the $Z^{\prime \prime }$ and $M^{\prime \prime }$ peaks tend to coincide, it indicates that the two tandem *R*‐*C* circuits become a single *R*‐*C* circuit without interfacial polarization. This means that the gap between two the peaks can represent the strength of interfacial polarization. We previously pointed out that the concentration of space charges and related relaxation is determined by dielectric mismatch (DM), which is defined as the difference in the relaxation time between grain and grain boundary:^[^ ^33^ ^]^

(7) $$\mathrm{DM}=\tau_{g}-\tau_{\mathrm{gb}}=\frac{\omega_{\mathrm{gb}}-\omega_{g}}{\omega_{g}\omega_{\mathrm{gb}}}\propto f_{\mathrm{gb}}-f_{g}$$

where subscripts g and gb represent the grain and grain boundary, respectively. *f* (associated with the angular frequency ω = 2π*f*) is the measurement frequency. Hence, the gap between the $Z^{\prime \prime }$ and $M^{\prime \prime }$ peaks, Δ*f* = |*f*_gb_ − *f*_g_|, virtually characterizes the interfacial polarization.

### 2.3 The Application of Interfacial Polarization

Based on Equation (7), the dielectric mismatch is mainly dominated by the resistance mismatch between grain and grain boundary, which paves the way for regulating interfacial polarization: modulating resistance mismatch. Being a defective phase, the resistance of the grain boundary is considered to be much higher than that grain. Therefore, the highly insulating CBST was used as a modifier to enhance the grain resistance. This can not only reduce the resistance mismatch thereby suppressing interfacial polarization, but also increase bandgap width, intrinsically improving *E*_b_ value.

### 2.4 Phase and Domain Structures

**Figure 3:**
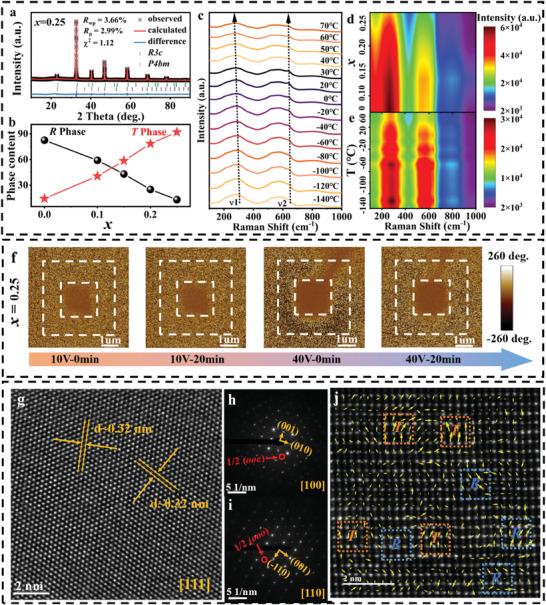
Phase and domain structures. a) Rietveld fitting to the XRD pattern of the 0.25CBST ceramic. b) Variation of *R*‐ and *T*‐phase upon the change of CBST content. c–e) Temperature‐dependent Raman spectra and variations with wavenumber determined by structure refinement. f) Electric field‐dependent PFM phase images after poling treatment and relaxation durations, g) HR‐TEM images, SAED patterns along h) [100]_c_ and i) [110]c axe, and j) HAADF‐STEM image along [110]_c_ axe of the *x* = 0.25 ceramic.

The crystal structure of the (BNT‐BT)‐*x*CBST ceramics was studied by XRD test. Figure S1a (Supporting Information) reveals a pure perovskite structure without a second phase. As the CBST content increases, the (200) diffraction peak gradually shifts toward a higher angle as seen in Figure S1b (Supporting Information). This is due to the larger Bi^3+^ ion (1.36 Å, CN = 12) and Na^+^ ion (1.39 Å, CN = 12) replaced by smaller Ca^2+^ ion (1.00 Å, CN = 12). To determine the variations of the *R*‐ and *T*‐phases with CBST content, Rietveld refinements were performed on the XRD patterns using the EXPGUI+GSAS package. *R*_wp_ and *R*_p_ are less than 5% (see Table S2, Supporting Information) indicating that refinement results are reasonable.^[^ ^20^ ^]^ The refinement results are shown in **Figures** **3**a and S1c–f (Supporting Information). The variation of the content of *R3c* (*R* phase) and *P4bm* (*T* phase) phases with CBST doping level is shown in Figure 3b. As the CBST content increases, the *T* phase content gradually increases and the *R* phase content gradually decreases. The *R* phase is a polar phase, while the *T* phase is a weakly polar phase, so the *R*/*T* ratio is important for an excellent performance. It can be demonstrated that the addition of CBST can effectively adjust the phase structure. Notably, the sample with *x =* 0.25 possesses an optimum *R*/*T* ratio of 14.5%, which is considered to be an appropriate proportion for excellent ESP.^[^ ^25^ ^]^ The sample is, therefore, selected for detailed investigation. To further elucidate the evolution of local structure contributing to the excellent stabilities of the 0.25CBST ceramic, we conducted Raman studies. Figure 3d shows the Raman spectra of the (BNT‐BT)‐*x*CBST ceramics. Raman spectra are divided into three main bands: (a) bands below 200 cm^−1^ associated with A‐site cations, (b) bands in the range 200–400 cm^−1^ associated with the B‐O vibration, and (c) bands associated with the TiO_6_ vibration corresponding to the 400–700 cm^−1^ band. With the increase of CBST content, the Raman vibration modes in the three bands become wider and more dispersed, which proves that the structural disorder of the samples is enhanced. To further study the evolution of the local structure in 0.25CBST, in situ Raman at various temperatures investigations were performed. The temperature‐dependent (−140 to 70 °C) Raman and corresponding contour map of peak position and intensity are shown in Figure 3c,e. It can be clearly seen that the 0.25CBST samples all have wider and smoother Raman peaks with the temperature from −140 to 70 °C, meanwhile the number of Raman peaks does not change, which suggests that the symmetry of the local structure of the 0.25 sample does not change over a wide temperature range. It is noteworthy that the vibration mode within 200–400 cm^−1^ wave number is mainly B‐O bond vibration, represented by *v*1.^[^ ^20^ ^]^ The vibration mode within 400–700 cm^−1^ wave number can be attributed to BO_6_ octahedron vibration, represented by *v*2. Figure 3e shows more visually the detailed changes in peak position and intensity for the *v*1 and *v*2 modes. As the temperature increases, the 0.25CBST sample exhibits a broadening of the Raman peak and a decrease in intensity, which proves that the 0.25CBST sample is highly localized and disordered, thereby enhancing the ESP.

To further investigate the reason for the excellent performance of 0.25CBST in relation to the local domain structure. The domain structure was directly observed by piezoresponse force microscopy (PFM). An electric field is applied to a 3 × 3 µm^2^ local area (white dotted box). Figure 3f shows the PFM phase images of the 0.25CSBT sample at different electric fields after different poling treatment times. The phase contrast of the 0.25CSBT sample is not easily detected under 10 V, indicating that the sample has small ferroelectric domains. This also verifies that the 0.25CSBT ceramic has narrow *P‐E* loops under low‐medium electric fields. However, when the voltage was increased to 40 V, the phase contrast of the 0.25CBST sample was significant, indicating that the ferroelectric domains were very large at this time. When the applied voltage is removed after 20 min, it returns to the original state. This property confirms that the 0.25CSBT ceramic has larger *P*_max_ and smaller *P*_r_, indicating the existence of highly dynamic PNRs.^[^ ^10^ ^]^

In order to observe the phase structure and PNRs, the 0.25CSBT sample was studied by transmission electron microscope (TEM). No significant ferroelectric domains were observed in bright field mode image in Figure S2 (Supporting Information), which explains that the introduction of CBST breaks the ferroelectric domain and forms PNRs. According to HR‐TEM image in Figure 3g, the crystal plane spacing of the 0.25CSBT sample is 0.32 nm. Meanwhile, it can be found that the sample has uniform lattice stripes, indicating that CBST is fully incorporated into the NBT‐BT matrix lattice. The selected area electron diffraction (SAED) images along the ribbon axes of [100]_c_, and [110]_c_ are shown in Figure 3h,i. The typical 1/2 (*ooe*) superlattice diffraction points (corresponding to the *P4bm* phase) associated with the tilting of the in‐phase a^0^a^0^c^+^ oxygen octahedron can be observed along the [100]_c_ ribbon axis. And the typical 1/2 (*ooo*) superlattice diffraction points (corresponding to the *R3c* phase) associated with the tilting of the inverted a^−^a^−^a^−^ oxygen octahedron also can be observed along the [110]_c_ ribbon axis. These also effectively validate the results of the XRD refinements. The expected polymorphic PNRs were detected accurately by an aberration‐corrected scanning transmission electron microscope (STEM) with atomic resolution in detail. Atomic‐resolution HAADF STEM polarization vector images of the 0.25CBST ceramic along [110]_c_ are shown in Figure 3j, as indicated by the results of the two‐dimensional Gaussian peak fitting.^[^ ^34^, ^35^ ^]^ The results indicate that by using different polarization vectors with different directions and amplitudes, *R* and *T* phases can be effectively distinguished. This multiphase coexistence of PNRs can efficiently reduce the domain size for superior ESP.

### 2.5 Energy‐Storage Performance of the (BNT‐BT)‐*x*CBST Ceramics

**Figure 4:**
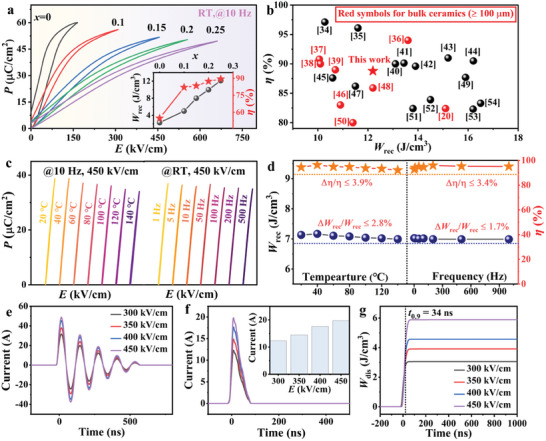
Energy‐storage performance of the (BNT‐BT)‐xCBST samples. a) The room temperature unipolar *P‐E* loops of the (BNT‐BT)‐*x*CBST ceramics measured at their *E*_b_ and 10 Hz. Insets are the calculated *W*_rec_ and η values under *E*_b_. b) Comparison of the *W*_rec_ (>10 J cm^−3^) and *η* (> 80%) between this work and other ceramics. c) *P‐E* loops of the 0.25CBST ceramic under different temperatures and frequencies. d) The calculated *W*_rec_ and *η* as a function of temperature and frequency. e–g) Underdamped and overdamped discharge properties of the 0.25CBST ceramic.

**Figure** **4**a displays the unipolar *P‐E* loops of the (BNT‐BT)‐*x*CBST ceramics tested under high fields up to their *E*_b_. The loop gradually becomes slender, and *P*_m_ decreases with increasing the CBST content. This is characteristic of relaxor ferroelectric materials, indicating that the addition of CBST breaks the long‐range ferroelectric domains, yielding short‐range polar nanoregions.^[^ ^10^, ^20^ ^]^ As the CBST content (*x*) increases from 0 to 0.25, the *E*_b_ values are found to be 165, 310, 450, 555, and 660 kV cm^−1^. This confirms that the addition of CBST notably improves the breakdown strength, which is vital for an excellent ESP. The inset of Figure 4a presents the corresponding *W*_rec_ and *η*. It is found that the *W*_rec_ increase from a very low value of 2.3 J cm^−3^ for 0CBST remarkably increases to an ultrahigh value of 12.2 J cm^−3^ in 0.25CBST. The *η* drastically increases from 54.7% for 0CBST to a large value of 88.8% for 0.25CBST. Finally, an ultrahigh *W*_rec_ of 12.2 J cm^−3^ with *η* = 88.8% is achieved in the 0.25CBST ceramic. Figure S3 (Supporting Information) shows the *P‐E* curves of the 0.25CBST ceramic measured by increasing *E* from 100 to 660 kV cm^−1^. The derived *W*_rec_ and *η* as a function of *E* is shown in the inset of Figure S3 (Supporting Information). The increase in *E* leads to a significant increase in *W*_rec_ from a very low value of 0.74 J cm^−3^ at 100 kV cm^−1^ to a value of 12.2 J cm^−3^ at 660 kV cm^−1^. This superior ESP is comparable to the best ESP values with *W*_rec_ > 10 J cm^−3^ and *η* > 80% reported so far in Pb‐free dielectric materials,^[^ ^20^, ^34^, ^35^, ^36^, ^37^, ^38^, ^39^, ^40^, ^41^, ^42^, ^43^, ^44^, ^45^, ^46^, ^47^, ^48^, ^49^, ^50^, ^51^, ^52^, ^53^, ^54^ ^]^ as reflected in Figure 4b. It is worth emphasizing that this superior ESP is obtained in bulk ceramics with *d* ≥ 100 µm. To date, only a few bulk ceramics have achieved *W*_rec_ higher than 12 J cm^−3^, as highlighted by red symbols in Figure 4b.

Good stability of dielectric energy storage ceramics is crucial for practical applications, especially in various harsh environments.^[^ ^24^ ^]^ So the frequency, temperature, and cycle stabilities of the 0.25CBST sample were tested at 450 kV cm^−1^. The results shown in Figure 4c,d and Figure S4a (Supporting Information) reveal that when the temperature changes from 20 to 140 °C, the P‐E loops of the material remain almost unchanged. The Wrec basically remains ≈7.1 J cm^−3^, and the change rate of the Wrec is much less than 2.8%. In addition, the η decreases from 94.6% (20 °C) to 92.3% (140 °C), with a change rate of less than 3.9%. The P‐E loops of the material also remain almost unchanged when the frequency range of 1–500 Hz, the Wrec basically remains ≈7.1 J cm^−3^, and the change rate of the Wrec is much less than 1.7%. In addition, the η decreases from 94.6% (1 Hz) to 92% (500 Hz), with a change rate of less than 3.4%. Meanwhile, when the cycle numbers change from 1 to 5000, the P‐E loops of the material hardly change in Figure S4b (Supporting Information). The Wrec and η maintain stable values ≈7.1 J cm^−3^ and 94%, respectively. This can be attributed to the polarization response process dominated by the growth and orientation of PNRs under the electric field response.^[^ ^45^ ^]^

In parallel, the charging and discharging properties of energy storage ceramics are equally important in practical applications.^[^ ^34^ ^]^ The underdamped charging properties of the 0.25CBST under different *E* were shown in Figure 4e. As the electric field increases, the peak current (*I*) increases significantly, and notably, an ultra‐high current value of 48.8 A was obtained at 450 kV cm^−1^. Current density (*C*_D_) and power density (*P*_D_) are:^[^ ^34^ ^]^

(8) $$C_{D}=I_{\max}/S$$

(9) $$P_{D}=EI_{\max}/2S$$

where *I*_max_ and *S* denote the maximum current and the area of the electrode, respectively. In Figure S5a (Supporting Information), as *E* increases, the values of *C*_D_ and *P*_D_ increase monotonically, leading to the *C*_D_ and *P*_D_ reaching high values of 1554 A cm^−2^ and 349 MW cm^−3^ under 450 kV cm^−1^, respectively. When the temperature changes from 20 to 140 °C, the peak current value of the 0.25CBST remains almost unchanged under 450 kV cm^−1^ in Figure S5b (Supporting Information). Meanwhile, the deduced values of the *C*_D_ and *P*_D_ are shown in Figure S5c (Supporting Information). It is obvious to notice that both *C*_D_ and *P*_D_ show very small variations. This result evidences the sample has great potential for energy storage applications.

Figure 4f and its inset display overdamped discharge electric current‐time (*I*‐*t*) curves under different *E*. The *I*_max_ increases significantly with the electric field increases. The overdamped discharging energy density (*W*_dis_) is also a key performance parameter, defined formula as:^[^ ^55^ ^]^

(10) $$Wdis=R\int i^{2}\left(t\right)dt/V$$

where *R* is the load resistance (200 Ω), and *V* is the bulk sample's volume.^[^ ^49^, ^50^ ^]^ The calculated *W*_dis_ based on Equation (10) is shown in Figure 4g. A high *W*_dis_ value of 5.8 J cm^−3^ was obtained at an electric field of 450 kV cm^−1^, which is lower than *W*_rec_ because of the energy loss due to defects in the sample and external loads in the circuit. In addition, a fast charge/discharge time of *t*_0.9_ = 34 ns was obtained for the sample (*t*_0.9_ indicates the time taken for 90% of the total energy density to be discharged). The above results evidence that the 0.25CBST sample exhibits superior ESP along with excellent temperature/frequency, and good charging and discharging performance, indicating that the sample meets the requirements of practical pulsed applications.

### 2.6 Evolution of Interfacial Polarization in the (BNT‐BT)‐*x*CBST Ceramics

**Figure 5:**
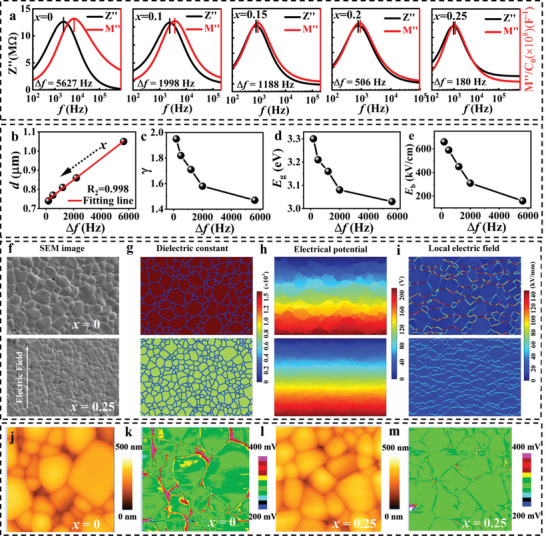
Evolution of interfacial polarization. a) The spectroscopic plots of impedance and modulus for each sample at 530 °C. Inset shows the gap, Δ*f*, between the $Z^{\prime \prime }$ and $M^{\prime \prime }$ peaks as a function of the CBST content. b–e) The values of *d*, *γ*, *E*_g_, and *E*_b_ as a function of Δ*f*. f) SEM images of *x* = 0, and 0.25 ceramics, based on which grain and grain boundary patterns are extracted. g) The dielectric constant, h) electric potential, and i) local electric field distributions. j,l) Surface morphology and k,m) surface potential distribution of *x* = 0 and 0.25 samples.

To determine whether the excellent ESP achieved in this work is related to interfacial polarization, we performed impedance and electric modulus tests on each sample in Figure S6 (Supporting Information). The spectroscopic plots of $Z^{\prime \prime }$ and $M^{\prime \prime }$ were displayed in **Figure** **5**a. The $Z^{\prime \prime }$ and $M^{\prime \prime }$ peak‐gap, Δ*f*, decreases from 5627 Hz for the *x* = 0 sample to a very small value of 180 Hz for the *x* = 0.25 sample. It is worth emphasizing that Δ*f* → 0 means that the interfacial polarization tends to disappear, and the electric field tends to be evenly distributed. This finding firmly demonstrates that the addition of CBST effectively suppresses interfacial polarization.

As mentioned above, the excellent ESP in the *x* = 0.25 sample is related to the ultrahigh *E*_b_ value. Besides interfacial polarization, factors such as grain size and bandgap width can also influence the *E*_b_. To investigate the variations in mean grain size with *x*, Figure S7 (Supporting Information) presents the SEM images of the (BNT‐BT)‐*x*CBST ceramics. The mean grain size can be deduced from the grain‐size statistical distribution mappings shown as insets in Figure S7 (Supporting Information). With the CBST content increases, the average grain size obviously decreases from 1.05 to 0.74 µm. This fact can be attributed to CBST entering the BNT‐BT matrix, effectively inhibiting grain growth and making the grains more uniform. The reduction in grain size helps to increase energy storage density. Interestingly, as seen in Figure 5b, there is a perfect linear relationship between *d* and Δ*f*. Linear fitting yields the figure of merit *R* ^2^ = 0.998. The two points of *x* = 0.20 and 0.25 deviate obviously from linearity. This is because, as aforementioned, when Δ*f* is very close to zero, the sample tends to a critical state similar to a phase transition. This result demonstrates that the reduction in grain size is directly related to the suppression of interfacial polarization.

It is worth noting that the suppression of interfacial polarization can lead to a decrease in the dielectric constant. To corroborate this inference, Figure S8a–e (Supporting Information) shows the variations of dielectric constant (ε′) and loss tangent (tanδ) with temperature for the (BNT‐BT)‐*x*CBST ceramics. The dielectric peak obviously shifts to low temperature with increasing CBST content, featuring typical relaxor behavior characterized by phase transition diffusion and frequency diffusion. The peak shifts to a temperature lower than 50 °C in the 0.25CBST sample. Figure S8f (Supporting Information) compares the ε′(*T*) curves of all the samples measured at 1 MHz, clearly confirming that the addition of CBST significantly reduces the dielectric constant and improves thermal stability. The relaxor factor (*γ*), is deduced from the modified Curie‐Weiss law formula.^[^ ^56^ ^]^

(11)

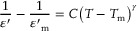




The fitting results of the ε′(*T*) curves recorded under 1 MHz based on Equation (11) for all the samples are presented in Figure S9 (Supporting Information). *γ* grows from 1.47 to 1.95 with increasing *x* from 0 to 0.25, further confirming that CBST addition leads to enhanced traversal relaxor behavior. A high *γ* value of 1.95 is obtained in the *x* = 0.25 sample, indicating superior relaxor behavior. This result confirms the enhancement of the relaxor behavior as Δ*f* decreases, as shown in Figure 5c.

It is well‐known that the *E*_b_ value is intrinsically related to the forbidden bandwidth *E*_g_.^[^ ^25^ ^]^ Therefore, the UV–vis absorption spectroscopy results and the corresponding Tauc plots are shown in Figure S10a,b (Supporting Information), respectively. The results show that *E*_g_ gradually increases from 3.03 to 3.31 eV as the content of CBST increases from 0 to 0.25. The enhancement in *E*_g_ can be ascribed to the fact that the highly insulating CBST increases the bandwidth of the solid solution. Once again, we note that the bandgap (Figure 5d) and hence the breakdown strength (Figure 5e) notably increases as the interface polarization tends to disappear (i.e., Δ*f* → 0). Excitingly, the important factors of *d*, *γ*, and *E*_g_ that affect *E*_b_ are all linked to interfacial polarization. Suppressing the interfacial polarization (Δ*f* → 0) leads to a remarkable refinement of *d* and drastic enhancement in *γ* and *E*_g_. All these changes are beneficial for achieving superior ESP, suggesting that these parameters may be related to the physics behind the interfacial polarization.

As mentioned, the disappearance of interface polarization leads to a more evenly distributed electric field. Numerical simulations of the electrical properties of two representative samples (*x* = 0 and 0.25) were carried out by finite element software (COMSOL).^[^ ^20^, ^22^ ^]^ These electrical properties results were pictured in Figure 5g–i. Based on the SEM images (Figure 5f), the grain becomes smaller and more uniform, and the dielectric constant decreases, making the transition between the grain boundaries milder in the 0.25CBST sample. The simulated potential distribution after 100 kV cm^−1^ electric field is shown in Figure 5h,i. The potential distribution of the 0.25CBST sample is much more uniform than that of the 0CBST sample. Meanwhile, the red high LEF area on the grain boundary decreases and the electric field distribution tends to be more uniform in the 0.25CBST sample. The COMSOL simulation results clearly indicate that suppressing interfacial polarization promotes uniform distribution of electric field, which is beneficial for improving *E*_b_.

Kelvin probe force microscopy (KPFM) was further used to directly verify the results of the COMSOL simulation. The surface potential distribution and topography of the poled samples with an applied voltage of 10 V were collected. The result for the *x* = 0 sample, as shown in Figure 5j,k, reveals a significant contrast difference in surface potential between grains and grain boundaries. This contrast is caused by the substantial difference in electrical resistance between the grains and the grain boundaries, thereby confirming strong interfacial polarization in the sample. However, a uniform surface potential distribution between grains and grain boundaries was found in the *x* = 0.25 sample (Figure 5l,m). This finding firmly demonstrates that the suppression of the interfacial polarization enhances the homogeneity of the electric field, and clearly validates the COMSOL simulation results.

## 3 Conclusions

In conclusion, we investigated the physics behind interfacial polarization and how it can be utilized to achieve superior ESP in lead‐free ceramics. The frequency gap between the impedance and electric modulus peaks was suggested to be a feasible parameter to describe the interfacial polarization. And insulating dielectrics were proposed as suitable modifiers to tune the interfacial polarization. The effectiveness of interfacial polarization engineering is validated in (BNT‐BT)‐*x*CBST ceramics. Our results demonstrate that the addition of linear‐like dielectric CBST effectively suppresses interfacial polarization, leading to grain size refinement and enhancements in relaxor factor, bandgap, and breakdown strength. An ultrahigh *W*_rec_ of 12.2 J cm^−3^ and a large *η* of 88.8% were achieved in the (BNT‐BT)‐0.25CBST sample. Furthermore, the sample exhibits excellent temperature/frequency and long‐term stabilities, good charging and discharging performance. This work highlights that interfacial polarization engineering would be a facile strategy for designing dielectric ceramics with excellent ESP.

## 4 Experimental Section

### Sample Preparation

The ceramics of (BNT‐BT)‐*x*CBST with *x* = 0.0, 0.10, 0.15, 0.20, and 0.25 were synthesized through the solid‐state reaction method. The details of sample experimental preparation procedures of the ceramics are provided in the Supporting information.

### Electrical Property Measurements

To measure the electrical properties, gold electrodes with a diameter of 1 mm were sputtered on the surface of the polished sample. The *P‐E* curves were tested using a ferroelectric hysteresis measurement tester (MultiFerroic II, America) with a sample thickness of 0.12 mm. Charge‐discharge performance was characterized for 0.25CBST ceramic with a thickness of 0.12 mm and a gold electrode diameter of 2 mm, where the external load resistance was 200 Ω. After covering the polished sample surface with silver electrodes, the dielectric and impedance properties were tested using the Wayne Kerr 6500B impedance analyzer (Wayne Kerr, China). The ceramic sample surfaces were polished with polishing paste and cloth, and then the environment for thermal etching is to heat all samples at a rate of 10 °C min^−1^ to 1100 °C for 30 min. Finaly, the surface potential distribution of the samples was tested using a Kelvin Probe Force Microscope (KPFM, Hitachi 5500 m).

### Structure Characterizations

The crystal structure of the powder samples was characterized using an X‐ray diffractometer (Rigaku Smartlab Beijing Co, China). Rietveld refinements were carried out using the program GSAS software. The ceramic surface was polished and then the environment for thermal etching was to heat all samples at a rate of 10 °C min^−1^ to 1100 °C for 30 min. Raman spectroscopy (Renishaw in Via) was used to characterize the evolution of the local crystal structure. The 0.25CBST ceramic was cut into 10 um slices using Carl Zeiss Crossbeam 550L focused ion beam (FIB) and then tested under the JEOL JEM‐2100 instrument for bright field TEM, HR‐TEM, SEAD. HAADF‐STEM images were observed by a JEOL ARM 200 STEM with a spherical aberration corrector operated at 200 kV.

The evolution of electric breakdown paths and distribution of the local electric field of the (BNT‐BT)‐xCBST ceramics with x = 0.0 and 0.25 were analyzed using a finite element software COMSOL (See Supporting information for details). Before observing the grain distribution by scanning electron microscopy (Regulus 8230, Japan), the sintered ceramic surface needs to be sputtered with gold. The bandgap width of all the samples was measured by UV–vis spectroscopy (PerkinElmer Lambda). The domain structure of poled ceramics was characterized by PFM (Bruker Dimension). The applied electric field was 10–40 V.

## Conflict of Interest

The authors declare no conflict of interest.

## Supporting information



Supporting Information

## Data Availability

The data that support the findings of this study are available from the corresponding author upon reasonable request.
