# Supplementary material for: Interfacial‐Polarization Engineering in BNT‐Based Bulk Ceramics for Ultrahigh Energy‐Storage Density
Source: Adv Sci (Weinh). 2024 Nov 8;11(48):2409113. doi: 10.1002/advs.202409113 (PMC11672256; doi:10.1002/advs.202409113)
Supplement: Supplementary file 1 — Supporting Information [file ADVS-11-2409113-s001.docx]

**Supporting information**

**Interfacial-polarization engineering in BNT-based bulk ceramics for ultrahigh energy-storage density**

Wenjun Cao^1^, Li Li^1^, Kun Chen^1^, Xuecen Huang^1,2^, Feng Li^3^, Chunchang Wang^1^*, Jun Zheng^3^*, Xu Hou^4, 5^*, Zhenxiang Cheng^6^*

^1^Laboratory of Dielectric Functional Materials, School of Materials Science & Engineering, Anhui University, 230601 Hefei, China.

^2^School of Material and Chemical Engineering, Chuzhou University, 239000 Chuzhou, China

^3^Institute of Physical Science and Information Technology, Anhui University, 230601 Hefei, China.

^4^Department of Industrial and Systems Engineering, Research Institute for Advanced Manufacturing, The Hong Kong Polytechnic University, Hung Hom, Kowloon, Hong Kong, China.

^5^Hong Kong Polytechnic University Shenzhen Research Institute, 518060 Shenzhen, China.

^6^Institute for Superconducting and Electronic Materials, Faculty of Engineering and Information Sciences, University of Wollongong, North Wollongong, NSW 2500, Australia.

*Corresponding author, E-mail: ccwang@ahu.edu.cn (WANG), jzheng@ahu.edu.cn (ZHENG), emhouxu@zju.edu.cn (HOU), cheng@uow.edu.au (CHENG).

**Table S1** Lead-free ceramics with *W*_rec_ exceeding 10 J/cm^3^

| **Composite** | **Thickness**(μm) | ***E*_b_**  (kV/mm) | ***W*_rec_** (J/cm^3^) | ***η*** (%) | **Ref.** |
| --- | --- | --- | --- | --- | --- |
| 0.76NaNbO_3_-0.24(Bi_0.5_Na_0.5_)TiO_3_ | 150 | 68 | 12.2 | 69 | 1 |
| (Na_0.91_Bi_0.09_)(Nb_0.94_Mg_0.06_)O_3_ | 80-100 | 78.3 | 10.9 | 83 | 2 |
| [(K_0.2_Na_0.8_)_0.8_Li_0.08_Ba_0.02_Bi_0.1_]  (Nb_0.68_Sc_0.02_Hf_0.08_Zr_0.1_Ta_0.08_Sb_0.04_)O_3_ | 60-100 | 74 | 10.06 | 90.8 | 3 |
| 0.9NaNbO_3_-0.1BiFeO_3_ | 60-80 | 99.5 | 18.5 | 78.7 | 4 |
| 0.62Bi_0.9_La_0.1_FeO_3_-0.3Ba_0.7_Sr_0.3_TiO_3_-  0.08K_0.5_Na_0.5_NbO_3_ | 50 | 63 | 13.9 | 89.6 | 5 |
| (Bi_0.5_Na_0.5_)TiO_3_-0.30SrTiO_3_-0.2(Sc_0.5_Ta_0.5_) ^4+^ | 100 | 59 | 12.2 | 85.9 | 6 |
| 0.88NaNbO_3_-0.12(BiFeO_3_-BaTiO_3_) | 50 | 93 | 14.5 | 83.9 | 7 |
| 0.6Bi_0.9_La_0.1_FeO_3_-0.3Ba_0.7_Sr_0.3_TiO_3_-  0.1Na_0.5_Nb_0.85_Ta_0.15_O_3_ | 50 | 72 | 15.9 | 87.7 | 8 |
| (Bi_0.5_Ba_0.1_Sr_0.1_Ca_0.2_Na_0.1_)(Fe_0.5_Ti_0.3_Zr_0.1_Nb_0.1_)O_3_ | 70-100 | 66.4 | 13.3 | 78 | 9 |
| 0.85BaTiO_3_-0.15(Bi_0.5_Na_0.5_)(Zn_1/3_Nb_2/3_)O_3_ | 60-80 | 58 | 11.6 | 96.1 | 10 |
| Bi_0.5_Na_0.5_TiO_3_-BaTiO_3_-0.1Zn_1/3_Nb_2/3_ | 50 | 80 | 20.3 | 89.3 | 11 |
| 0.55BiFeO_3_-0.33BaTiO_3_-  0.12NaTaO_3_ | 50 | 58 | 13.44 | 90.14 | 12 |
| 0.88NaNbO_3_-0.12(0.55BiFeO_3_-  0.45SrTiO_3_) | 50 | 97 | 16.2 | 82.3 | 13 |
| 1/3BaTiO_3_-1/3(Bi_0.5_Na_0.5_)TiO_3_-  1/3NaNbO_3_ | 50-70 | 54 | 10.59 | 87.6 | 14 |
| 0.848(Na,K)(Sb,Nb)O_3_-0.012SrZrO_3_-  0.14(Bi_0.5_Na_0.5_)ZrO_3_ | 80 | 74 | 13.1 | 90 | 15 |
| 0.88NaNbO_3_-0.12(Bi_0.8_Sr_0.2_)(Fe_0.9_Nb_0.1_)O_3_ | 60-80 | 98.3 | 16.5 | 83.3 | 16 |
| 0.62(0.94Na_0.5_Bi_0.5_TiO_3_-0.06BaTiO_3_)-0.38Ca_0.7_La_0.2_TiO_3_ | 120 | 64 | 15.1 | 82.4 | 17 |
| 0.90NaNbO_3_-0.10Sr(Fe_0.5_Ta_0.5_)O_3_ | 50 | 79 | 11.5 | 86.2 | 18 |
| (Na_0.5_Bi_0.5_)(Ti_1/3_Fe_1/3_Nb_1/3_)O_3_ | 80 | 64.3 | 13.8 | 82.4 | 19 |
| Ba_0.82_Bi_0.12_TiO_3_ | 80-100 | 70 | 10.1 | 90 | 20 |
| 0.85(0.75Na_0.5_Bi_0.5_TiO_3_-0.25BaTiO_3_)-0.15BaZrO_3_ | 100-150 | 66 | 13.6 | 94 | 21 |
| 0.85(0.75Na_0.5_Bi_0.5_TiO_3_-0.25BaTiO_3_)-0.12NaNbO_3_ | 50-70 | 72 | 15.2 | 91 | 22 |
| Bi_0.2_Na_0.2_Ba_0.2_Sr_0.2_Ca_0.2_TiO_3_-Li_2_CO_3_ | 200 | 64 | 10.7 | 89 | 23 |
| (Bi_0.5_Na_0.5_)_0.7_Sr_0.3_TiO_3_-Bi(Mg_1/3_Ta_2/3_)O_3_ | 50 | 56 | 10.28 | 97.11 | 24 |
| Bi_0.25_Na_0.25_Ba_0.5_Ti_0.92_Hf_0.08_O_3_ | 50 | 80 | 16.21 | 90.5 | 25 |
| Ag_0.5_Na_0.5_NbO_3_ | 100-150 | 67 | 11.4 | 80 | 26 |
| (BNT-BT)-*x*CBST | 120 | 66 | 12.2 | 88.8 | This work |





**Figure S1** **a** XRD patterns of the (BNT-BT)-*x*CBST ceramics with *x* = 0-0.25. **b** Partially enlarged view of (200) Bragg peaks. **c-f** Rietveld fittings for the XRD patterns of the (BNT-BT)-*x*CBST ceramics.

**Table S2** Rietveld refinement parameters of the (BNT-BT)-*x*CBST ceramics.

| Samples | Phase composition | a (Å) | c (Å) | R_p_ (%) | R_wp_ (%) | χ^2^ |
| --- | --- | --- | --- | --- | --- | --- |
| *x*=0 | *R3c* 82.51%  *P4bm* 17.49% | 5.5208  5.5411 | 13.4902  3.9210 | 4.32 | 3.63 | 1.26 |
| *x*=0.10 | *R3c* 59.23%  *P4bm* 40.77% | 5.5020  5.5388 | 13.4612  3.9103 | 3.89 | 3.06 | 1.18 |
| *x*=0.15 | *R3c* 42.12%  *P4bm* 57.88% | 5.4910  5.5276 | 13.4361  3.9002 | 3.26 | 2.86 | 1.06 |
| *x*=0.2 | *R3c* 25.02%  *P4bm* 74.98% | 5.4845  5.5185 | 13.4126  3.8861 | 4.18 | 3.89 | 1.21 |
| *x*=0.25 | *R3c* 12.66%  *P4bm* 87.34% | 5.4721  5.5004 | 13.4008  3.8154 | 3.66 | 2.99 | 1.12 |


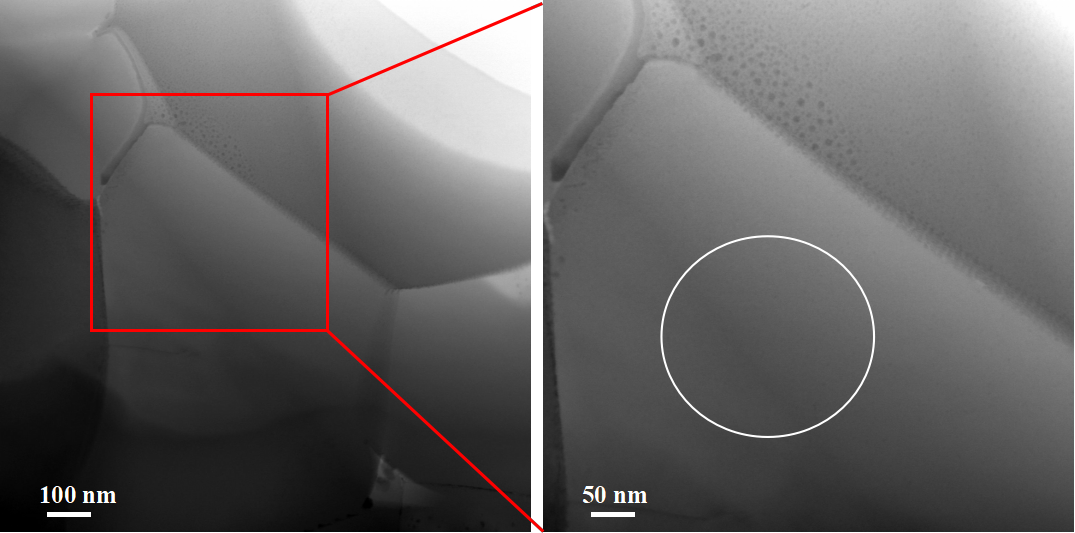


**Figure S2** Bright field mode image of the 0.25CBST ceramic, no ferroelectric domains were observed as highlighted by the white circle.


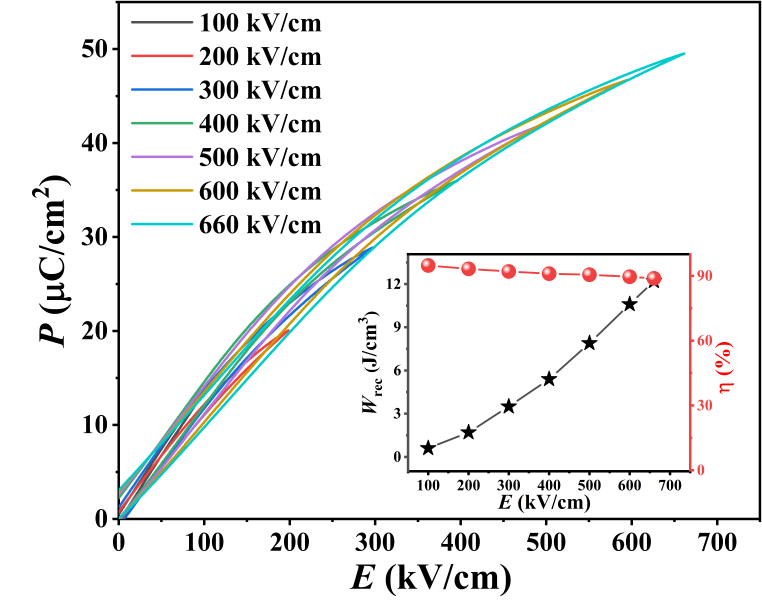


**Figure S3** *P-E* loops under different electric fields for the 0.25CBST ceramic. Inset shows the derived *W*_rec_ and *η* as a function of *E.*


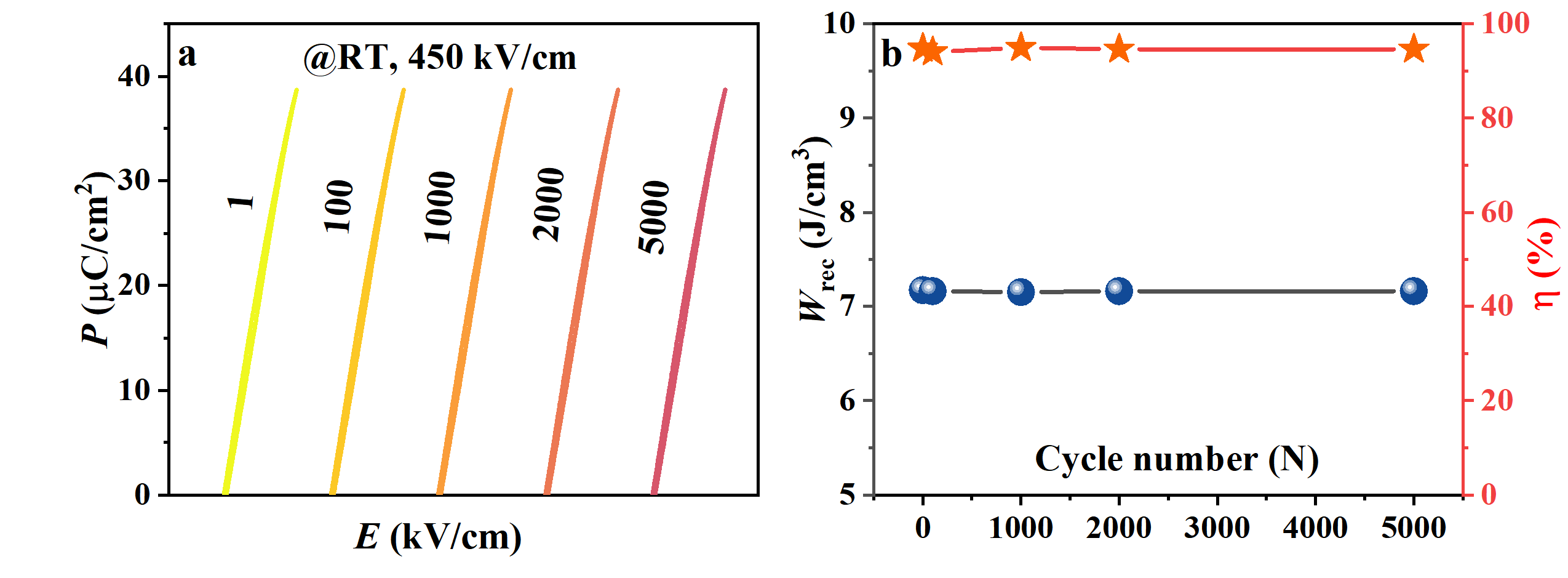


**Figure S4 a** *P-E* loops of the 0.25CBST ceramic under different cycle numbers. **b** The calculated *W*_rec_ and *η* as a function of cycle number.


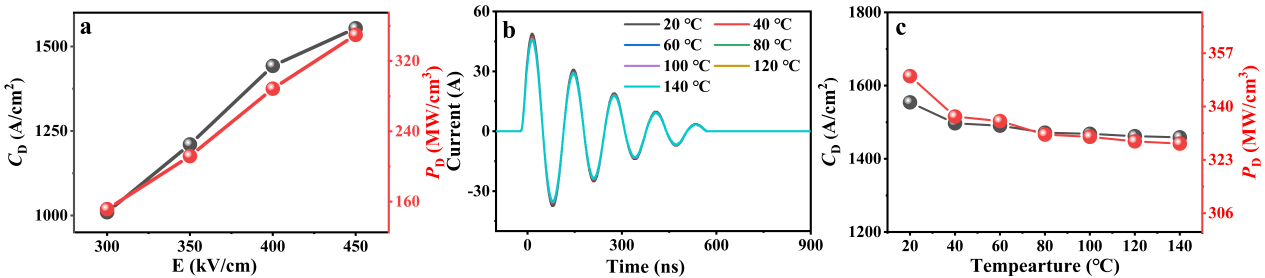


**Figure S5. a** the deduced values of *C*_D_ and *P*_D_ at various electric fields. **b** Undamped pulsed discharge current curves under various temperatures and **c** the deduced values of *C*_D_ and *P*_D_ for the 0.25CBST sample.


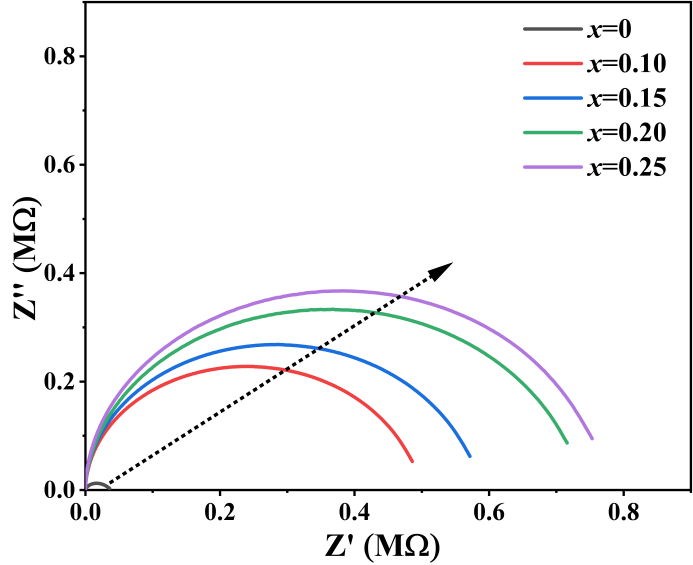


**Figure S6.** The complex impedance diagrams of all the samples recorded at 530℃.


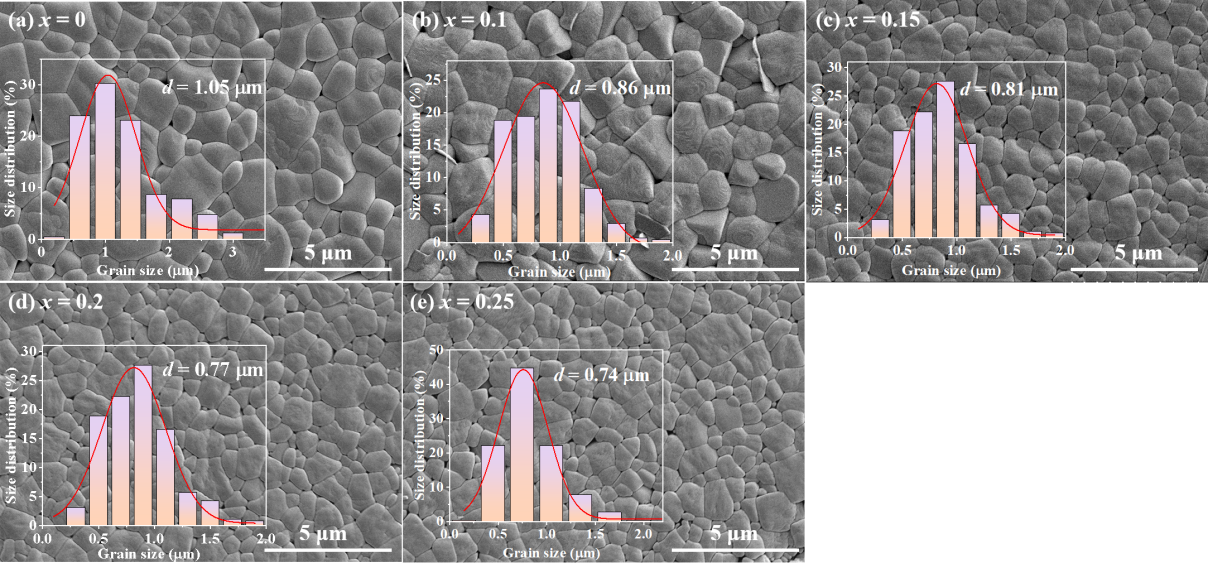


**Figure S7** SEM images corresponding grain size distributions of the (BNT-BT)-*x*CBST ceramics.


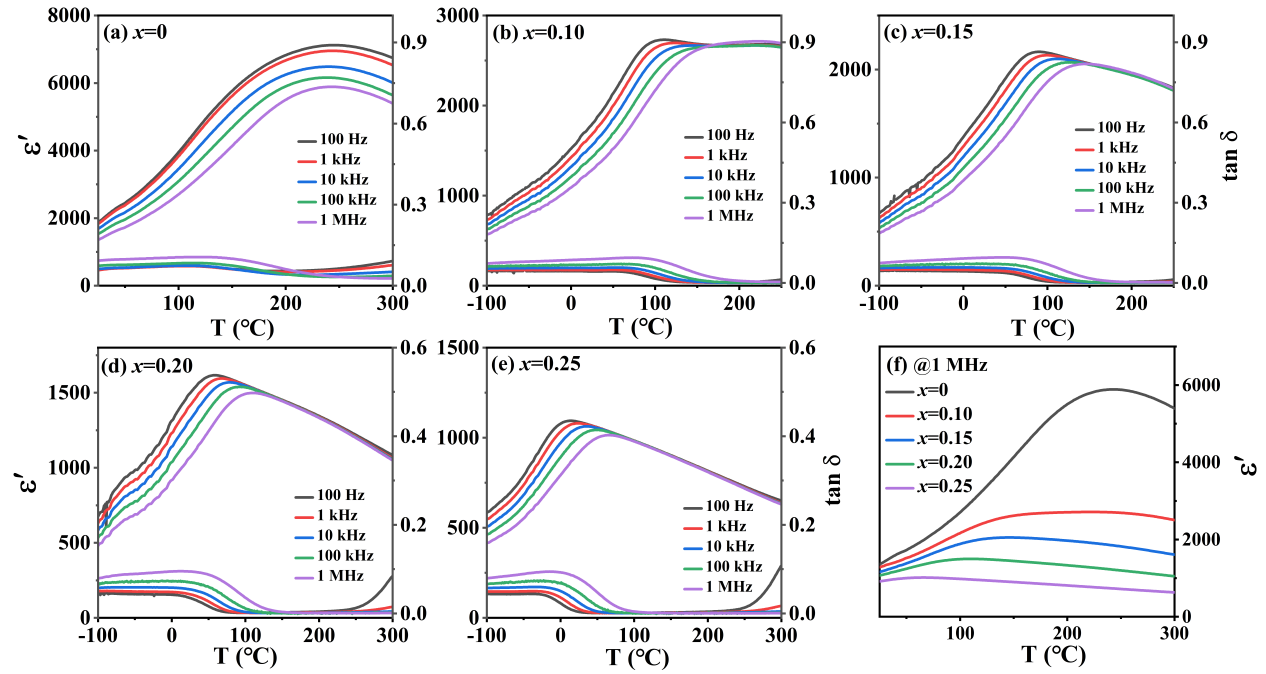


**Figure S8** Temperature dependence of and tanδ of **a** *x* = 0, **b** *x* = 0.10, **c** *x* = 0.15, **d** *x* = 0.20, **e** *x* = 0.25. **f** Temperature dependence of (1 MHz) of the (BNT-BT)-*x*CBST ceramics.

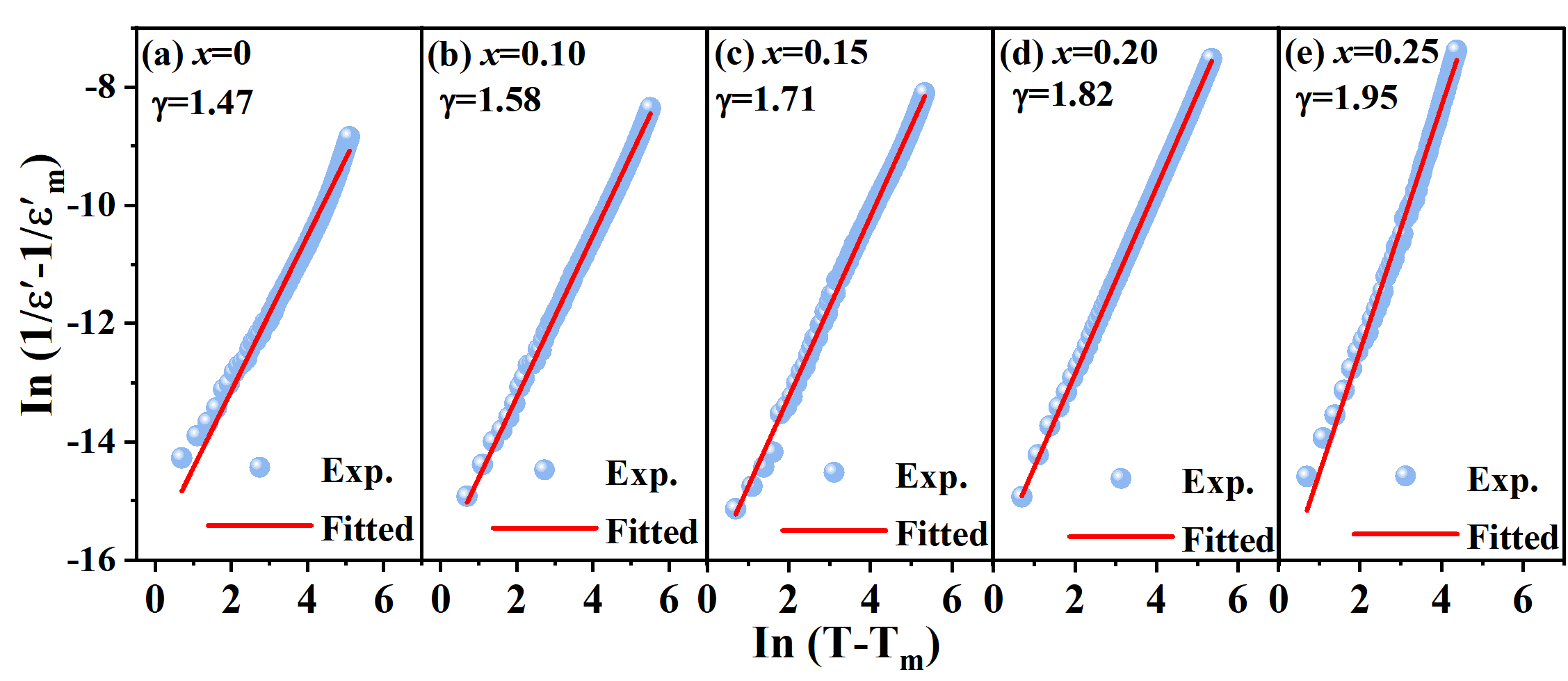


**Figure S9** Fitting results for the curves measured at 1 MHz based on the modified Curie-Weiss formula of the (BNT-BT)-*x*CBST ceramics.

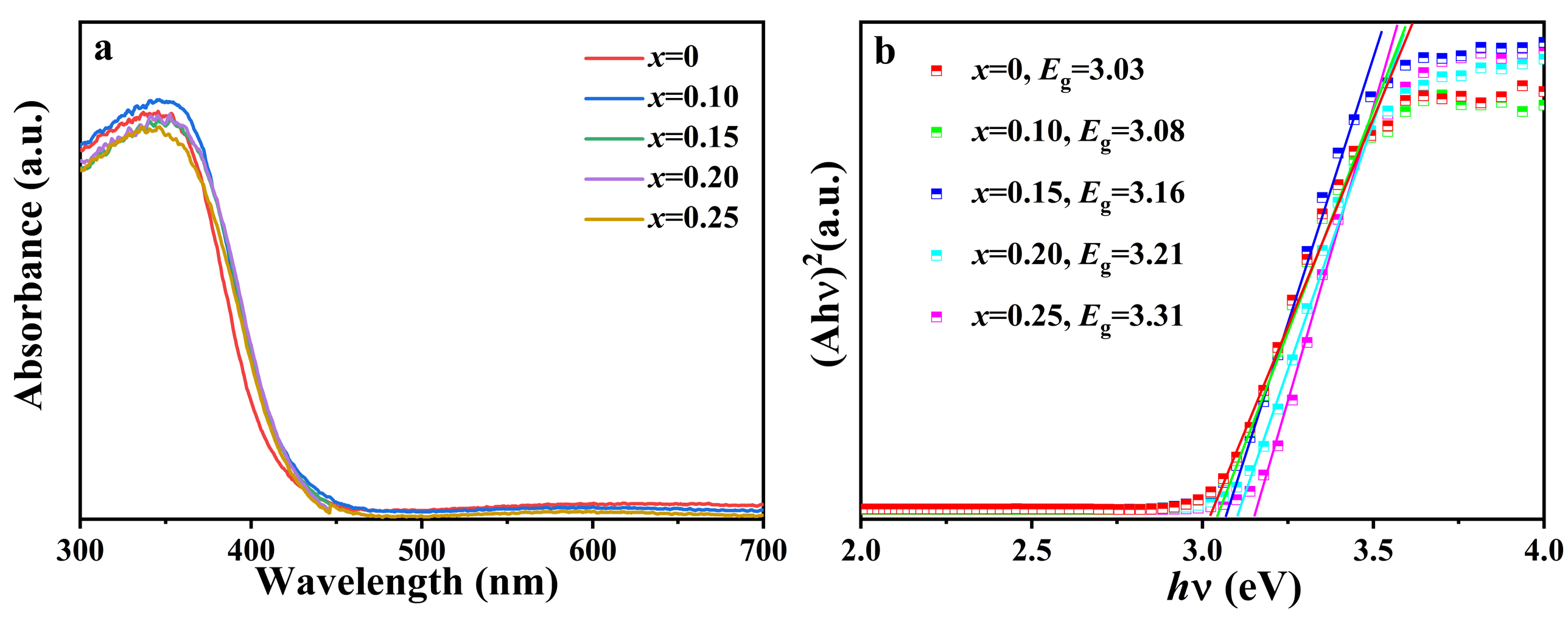


**Figure S10** **a** UV-vis absorption spectra and **b** the corresponding Tauc charts for the (BNT-BT)-*x*CBST ceramics.

**Experimental Section**

The raw powders of Bi_2_O_3_, Na_2_CO_3_, TiO_2_, BaCO_3_, CaCO_3_, SnO_2_ (all with purity ≥ 99%) were weighed. The weighed powders were ball milled in anhydrous ethanol under 300 rpm for 12 h. The BNT-BT powders were calcined at 850℃ for 6 h and the CBST powders were calcined at 1000℃ for 3 h in air. Two kinds of powders were remixed and then ball-milled again for 12 h. After dried, the mixture with 5 wt% PVA was pressed into pellets with 1 mm thickness and a diameter of 10 mm. After removing the organic binder at 600℃ for 2 h, the pressed disks were sintered at 1140-1180℃ for 3 h to obtain ceramic samples. Finally, the sintered ceramics were polished to 120 μm in thickness and then sputtered with gold electrodes (1 mm in diameter) for the energy-storage performance (ESP) measurements

**COMSOL Simulations**

In this simulation, we focused on the effect of inhomogeneous dielectric constants of grain and grain boundary on the local electric field distribution. The initial core-shell structures of grain and grain boundary for simulation were established based on the experimental results (SEM images). And the dielectric constant values of the grains and grain boundaries were assumed based on the mean grain size and overall permittivity value at 1 kHz. As shown in the Figure 5g, the red/green and blue colors represent the dielectric constants of the grain and grain boundary, respectively. The voltage was applied on the top boundary, whereas the bottom boundary was set to the ground. The up and down boundaries were speculated to be periodic. Finally electric potential and local electric field distributions is obtained by solving the electrostatic balance equation. The mesh style in this simulation is a mapped mesh with element size set to be ultra fine. The details of the phase field model for simulating the spatial distribution of electric breakdown path can be reached.^[27]^ The same design of core-shell structures established above is further employed for the simulation of electric breakdown strength. The grain cores and grain boundaries are extracted from the SEM images, and then assigned different permittivity values and breakdown energies. The external electric field is applied along the vertical direction of the sample slowly and finally kept as a large enough value for propagation of the breakdown path.

**References**

1. H. Qi, R. Zuo, A. Xie, A. Tian, J. Fu, Y. Zhang, S. Zhang, *Adv. Funct. Mater.* **2019***, 29*, 1903877.

2. J. Jiang, X. Meng, L. Li, J. Zhang, S. Guo, J. Wang, X. Hao, H. Zhu, S. Zhang, *Chem. Eng. J.* **2021**, *422*, 130130.

3. L. Chen, S. Deng, H. Liu, J. Wu, H. Qi, J. Chen, *Nat. Commun.* **2022**, *13*, 3089.

4. J. Jiang, X. Meng, L. Li, S. Guo, M. Huang, J. Zhang, J. Wang, X. Hao, H. Zhu, S. Zhang, *Energy Stor. Mater.* **2021**, *43*, 383.

5. T. Cui, J. Zhang, J. Guo, X. Li, S. Guo, Y. Huan, J. Wang, S. Zhang, *J. Mater. Chem. A* **2022**, *10*, 14316.

6. B. He, T. Ochirkhuyag, W. Feng, M. Liu, S. Liu, Z. Bao, C. Hu, Y. Zhong, D. Odkhuu, *J. Mater. Chem. A* **2023**, *11*, 14169-14179.

7. T. Pan, J. Zhang, Z. Guan, Y. Yan, J. Ma, X. Li, S. Guo, J. Wang, Y. Wang, *Adv. Electron. Mater.* **2022**, *8*, 2200793.

8. T. Cui, J. Zhang, J. Guo, X. Li, S. Guo, Y. Huan, J. Wange, S. Zhang, Y. Wang, *Acta Mater.* **2022**, *240*, 118286.

1. J. Wu, H. Tan, H. Qi, H. Yu, L. Chen, W. Li, J. Chen, *Small*, **2024**, *240*, 2400997.

10. L. Chen, T. Hu, X. Shi, H. Yu, H. Zhang, J. Wu, Z. Fu, He Qi, J. Chen, *Adv. Mater.* **2024**, *36*, 2313285.

11. Z. Sun, H. Liu, J. Zhang, H. Luo, Y. Yao, Y. Zhang, L. Liu, J. C. Neuefeind, J. Chen, *J. Am. Chem. Soc.* **2024**, *146*, 13476.

12. Z. Guan, Y. Yan, J. Ma, T. Pan, X. Li, S. Guo, J. Zhang, J. Wang, Y. Wang, *ACS Appl. Mater. Interfaces* **2022**, *14*, 44539-44549.

13. J. Ma, J. Zhang, J. Guo, X. Li, S. Guo, Y. Huan, J. Wang, S. Zhang, Y. Wang. *Chem. Mater.* **2022**, 34, 7313.

14. L. Chen, N. Wang, Z. Zhang, H. Yu, J. Wu, S. Deng, H. Liu, H. Qi, J. Chen, *Adv. Mater.* **2022**, *34*, 2205787.

15. A. Xie, J. Fu, R. Zuo, X. Jiang, T. Li, Z. Fu, Y. Yin, X. Li, S. Zhang. *Adv. Mater.* **2022**, *34*, 2204356.

16. J. Jiang, X. Li, L. Li, S. Guo, J. Zhang, J. Wang, H. Zhu, Y. Wang, S. Zhang, J. Materiomics **2022**, *8*, 295-301.

17. W. Cao, R. Lin, X. Hou, L. Li, F. Li, D. Bo, B. Ge, D. Song, J. Zhang, Z. Cheng, C. Wang, Adv. Funct. Mater. **2023**, *31*, 2301027.

18. T. Pan, J. Zhang, D. Che, Z. Wang, J. Wang, J. Wang, Y. Wang, *Appl. Phys. Lett.* **2023**, *122*, 072902.

19. L. Chen, H. Yu, J. Wu, S. Deng, H. Liu, L. Zhu, H. Qi, J. Chen, *Nano-Micro Lett.* **2023**, *15*, 65.

20. Z. Sun, J. Zhang, H. Luo, Y. Yao, N. Wang, L. Chen, T. Li, C. Hu, H. Qi, S. Deng, L. C Gallington, Y. Zhang, J. C Neuefeind, H. Liu, J. Chen, *J. Am. Chem. Soc.* **2023**, *145*, 6194-6202.

21. H. Liu, Z. Sun, J. Zhang, H. Luo, Q. Zhang, Y. Yao, S. Deng, H. Qi, J. Liu, L. C. Gallington, J. C. Neuefeind, J. Chen, *J. Am. Chem. Soc.* **2023**, *145*, 11764-11772.

22. H. Liu, Z. Sun, J. Zhang, H. Luo, Y. Yao, X. Wang, H. Qi, S. Deng, J. Liu, L. C Gallington, Y. Zhang, J. C Neuefeind, J. Chen, *J. Am. Chem. Soc.* **2023**, *145*, 19396-19404.

23. J. Guo, H. Yu Y. Ren, H. Qi, X. Yang, Y. Deng, S. Zhang, J. Chen, *Nano Energy* **2023**, *112*, 108458.

24. D. Li, D. Xu, W. Zhao, M. Avdeev, H. Jing, Y. Guo, T. Zhou, W. Liu, D. Wang, D. Zhou, *Energy Environ. Sci.* **2023**, *16*, 4511.

25. H. Luo, Z. Sun, J. Zhang, H. Xie, Y. Yao, T. Li, C.Lou, H. Zheng, N. Wang, S. Deng, L. Zhu, J. Liu, J. C. Neuefeind, M. G. Tucker, M. Tang, H. Liu, J. Chen, *J. Am. Chem. Soc.* **2024**, *146*, 460.

26. L. Chen, C. Zhou, L. Zhu, H. Qi, J. Chen, *Small* **2024**, *20*, 2306486.

27. J. Huang, X. Hou, S. Gao, Y. Zhou, H. Huang, Y. He, Q, Zhang, Journal of Materials Chemistry A, **2022**. *10*, 16337.
